# Large-Scale Growth of Tubular Aragonite Whiskers through a MgCl_2_-Assisted Hydrothermal Process

**DOI:** 10.3390/ma4081375

**Published:** 2011-08-08

**Authors:** Minyan Ren, Changyin Dong, Changhua An

**Affiliations:** 1 School of Petroleum Engineering, China University of Petroleum, Qingdao, Shandong 266555, China; E-Mails: anchh17@gmail.com (M.R.); dongcy@upc.edu.cn (C.D.); 2 State Key Laboratory of Heavy Oil Processing and College of Science, China University of Petroleum, Qingdao, Shandong 266555, China

**Keywords:** crystal morphology, whiskers, CaCO_3_, growth from solutions

## Abstract

In this paper, we have developed a facile MgCl_2_-assissted hydrothermal synthesis route to grow tubular aragonite whiskers on a large scale. The products have been characterized by powder X-ray diffraction (XRD), optical microscopy, and scanning electronic microscopy (SEM). The results show the as-grown product is pure tubular aragonite crystalline whiskers with a diameter of 5–10 μm and a length of 100–200 μm, respectively. The concentration of Mg^2+^ plays an important role in determining the quality and purity of the products. Furthermore, the method can be extended to fabricate CaSO_4_ fibers. The high quality of the product and the mild conditions used mean that the present route has good prospects for the growth of inorganic crystalline whiskers.

## 1. Introduction

As an important inorganic material, calcium carbonate (CaCO_3_) has been widely used as fillers in paper making, plastics, rubbers and coating due to its low price and richness in the world [1,2]. However, addition of such fillers beyond a certain level sometimes causes some problems such as reduced paper (or plastic, rubber) strength and stiffness. Crystalline whiskers with high aspect ratio, excellent mechanical properties, and perfect structures represent an ideal class of candidates as reinforcement in composite materials.

Aragonite, usually occurring in the form of a needle-like crystal, has attracted great interest owing to its great demand for the improvement of mechanical properties of polymer materials. For example, Shang and coworkers found that whisker aragonite performed better than calcite as fillers for polyvinyl alcohol and polypropylene composites [3]. Therefore, many approaches have been developed to synthesize aragonite whiskers. Inspired by the biomineralization processes, scientists have attempted to fabricate CaCO_3_ crystals with different morphologies and properties and have investigated the formation mechanisms [4,5,6]. Water-soluble additives such as metal ions [7,8], anion surfactants [9,10], polymers [11,12], and biomolecules [13] were introduced into the reaction systems to control the shapes of CaCO_3_ through specific interactions between the faces of the growing crystals and additives. The carbonation process, in which CO_2_ gas is bubbled through aqueous slurry of calcium hydroxide [13,14], is also often used to grow aragonite fibers in terms of environmental preservation and the effective use of mineral resources. However, the shape-control and modification of crystals is difficult, resulting in the limitation of the applicability of the method. Furthermore, granular calcite is usually easily co-produced. Recently, Yu and coworkers [15] have developed an ethanol/water system to realize polymorph discrimination of CaCO_3_ in which aragonite nanorods can be obtained by adjusting the ratio of solvents. Hou’s group [16] also produced aragonite rods in water/pyridine solution under solvothermal conditions. Unfortunately, these methods require a long time to get the desired product. Developing a facile approach to grow high quality aragonite whiskers under mild conditions still remains a challenge.

Herein, we have developed a convenient hydrothermal route with the assistance of magnesium chloride (MgCl_2_) to grow pure aragonite tubular whiskers using urea ((NH_2_)_2_CO) and calcium chloride (CaCl_2_) as starting materials. The influencing factors such as concentration, time, and Mg^2+^ additive have been investigated. Microscopy images show that the length of whisker is up to several hundred micrometers. Moreover, the method can be extended to prepare CaSO_4_ fibers. The high quality of whiskers, environmental benignity and low-temperature used mean the present method is promising in industrial applications.

## 2. Results and Discussion

**Figure 1:**
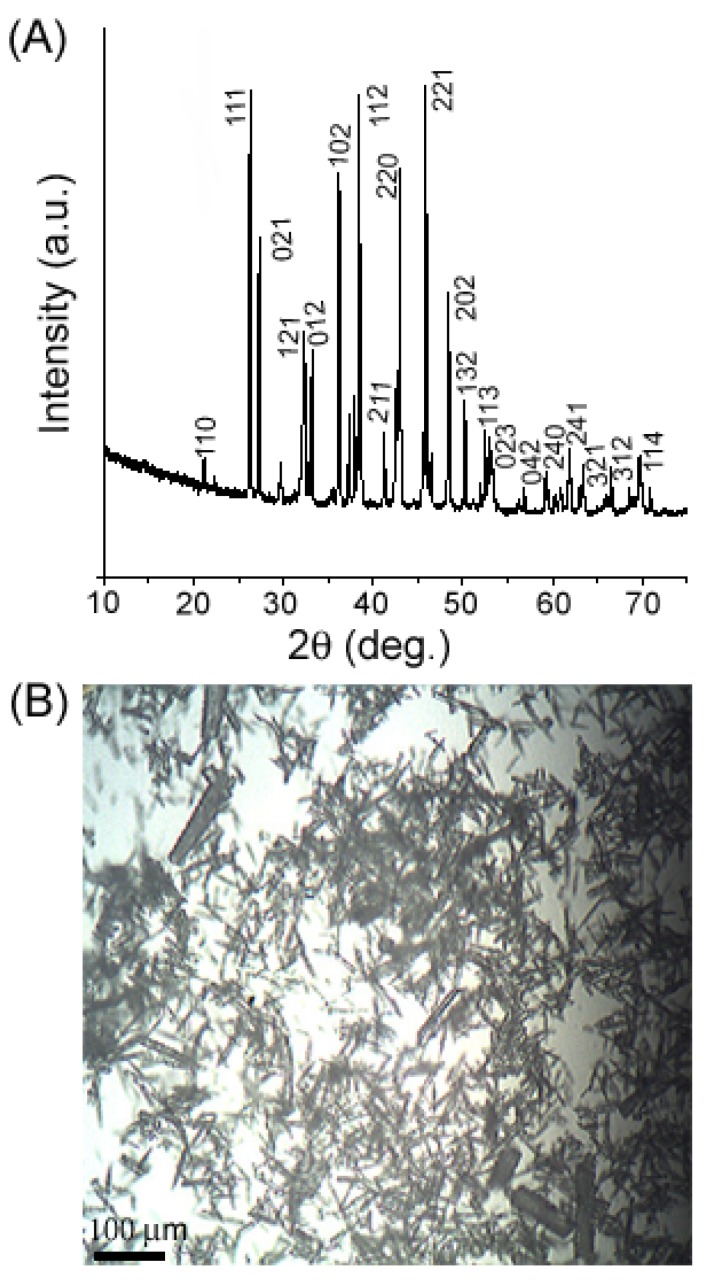
X-ray diffraction (XRD) pattern (**A**) and optical microscopy image (**B**) of the as-grown aragonite whiskers.

The phase and morphology of the products have been determined by XRD and optical microscopy and SEM. Figure 1 is the XRD pattern and optical microscopy image of the obtained aragonite whiskers. The XRD pattern shown in Figure 1(A) can be indexed to the orthorhombic phase CaCO_3_ with lattice constants *a* = 0.496 nm, *b* = 0.797 nm, *c* = 0.574 nm, consistent with the reported values (JCPDS PDF Card 76–0606). No calcite phase can be detected in the pattern, indicating that pure aragonite CaCO_3_ has been produced in the present process. The optical image in Figure 1(B) indicates that the product is composed of a large quantity of tubular aragonite whiskers with a diameter of 5–10 μm. The length of the whiskers is in a range of 100–200 μm.

It is known that high precipitation rate facilitates the formation of calcite phase because it hass greater entropy than aragonite [17]. Adjusting the nucleation rate of CaCO_3_ is essential to achieve pure aragonite crystals. In our system, the presence of appropriate magnesium ions inhibit the nucleation of calcite and thereby reducing carbonate activity below that required for calcite nucleation [18,19]. The main reactions involved in producing aragonite fibers can be expressed as follows:

(1) $${\left({\text{NH}}_{2}\right)}_{2}\text{CO }+{\text{ H}}_{2}\text{O}\to {\text{NH}}_{4}\text{OH }+{\text{ CO}}_{2}$$

(2) $${\text{CaCl}}_{2}+{\text{ 2NH}}_{4}\text{OH }+{\text{ CO}}_{2}\to {\text{ CaCO}}_{3}+{\;2\text{NH}}_{4}\text{Cl }+{\text{ H}}_{2}\text{O}$$

(3) $${\text{MgCl}}_{2}+{\;2\text{NH}}_{4}\text{OH}\to \text{Mg}{\left(\text{OH}\right)}_{2}+{\text{ 2NH}}_{4}\text{Cl}$$

**Figure 2:**
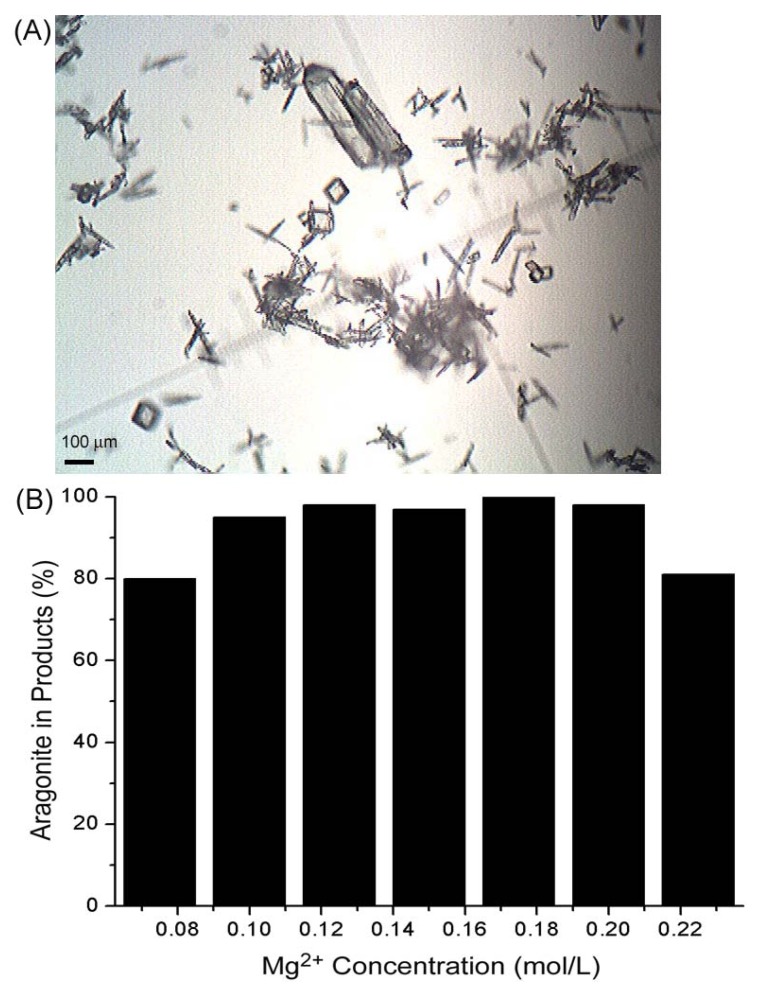
(**A**) Optical image of the product obtained with MgCl_2_ concentration of 0.075 mol∙L^−1^; (**B**) Variation of aragonite production with Mg^2+^ concentration when the Ca^2+^ is fixed at 0.36 mol∙L^−1^.

Similar cases are also found in oceanic conditions containing Mg^2+^ ions, in which metastable aragonite is precipitated first [18]. Kasuga and Ahn’s group [14,20] also demonstrated that Mg^2+^ played an important role in producing pure aragonite fibers in the carbonation process. Therefore, the purity of the aragonite can be controlled by changing the Mg^2+^ concentration. If excess Mg^2+^ is introduced, according to reaction (1) and (3), CO_2_ releases from reaction 1 will be fast and nucleation favors the formation of calcite. When little Mg^2+^ is used, the ability to control the nucleation rate of CaCO_3_ is weak and thus results in the occurrence of calcite. Here, we investigated the influence of the ratio of Ca^2+^/Mg^2+^ on the formation of aragonite tubular whiskers. It is found that the proper ratio of Ca^2+^/Mg^2+^ is from 63 to 8. For example, if Ca^2+^ concentration is kept at 0.36 mol∙L^−1^, MgCl_2_ can be introduced in a range of 0.1~0.2 mol∙L^−1^ and the resulting products are mainly composed of aragonite whiskers (Figure 2B). When 0.075 mol∙L^−1^ Mg^2+^ ions is used, the optical image (Figure 2A) shows that some calcite crystals with rhombus shape have been entrapped in the product On the other hand, excess Mg^2+^ (e.g., >0.2 mol∙L^−1^) also leads to the increase of calcite in the products (Figure 2B). Of course, detailed studies are necessary to give insights into understanding the role of Mg^2+^ in yielding tubular aragonite whiskers.

**Figure 3:**
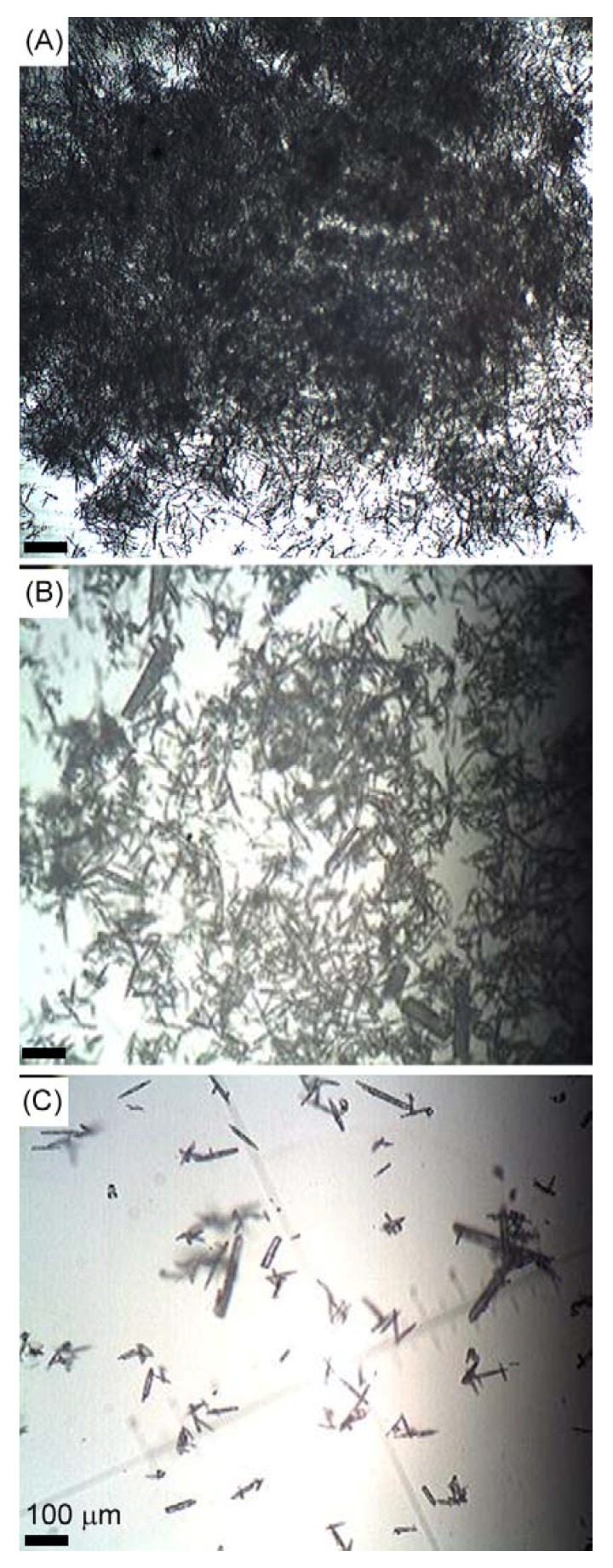
Optical microscopy images of the aragonite whiskers produced with different concentrations of starting materials (**A**) 1.44 mol∙L^−1^ CaCl_2_, (**B**) 0.72 mol∙L^−1^ CaCl_2_, (**C**) 0.06 mol∙L^−1^ CaCl_2_.

For real applications, the yields of the products in one-pot reaction are also very important. As shown in Figure 3, when CaCl_2_, (NH_2_)_2_CO, and MgCl_2_ are varied in an appropriate ratio, (*i.e.*, 0.06~1.44 mol∙L^−1^ for CaCl_2_, 0.18~4.32 mol∙L^−1^ for (NH_2_)_2_CO and 0.07~0.61 mol∙L^−1^ for MgCl_2_, respectively), the shape of the obtained aragonite crystals is well retained. It is notable that the yields of the whiskers are reduced as the feedstock concentration decreases. Moreover, high concentrations of feedstocks produce aragonite whiskers in high yields (Figure 3A). When 1.44 mol∙L^−1^ CaCl_2_ is used, the autoclave is full of aragonite whiskers without any liquid. This feature means the present method has good prospects for industrial application.

**Figure 4:**
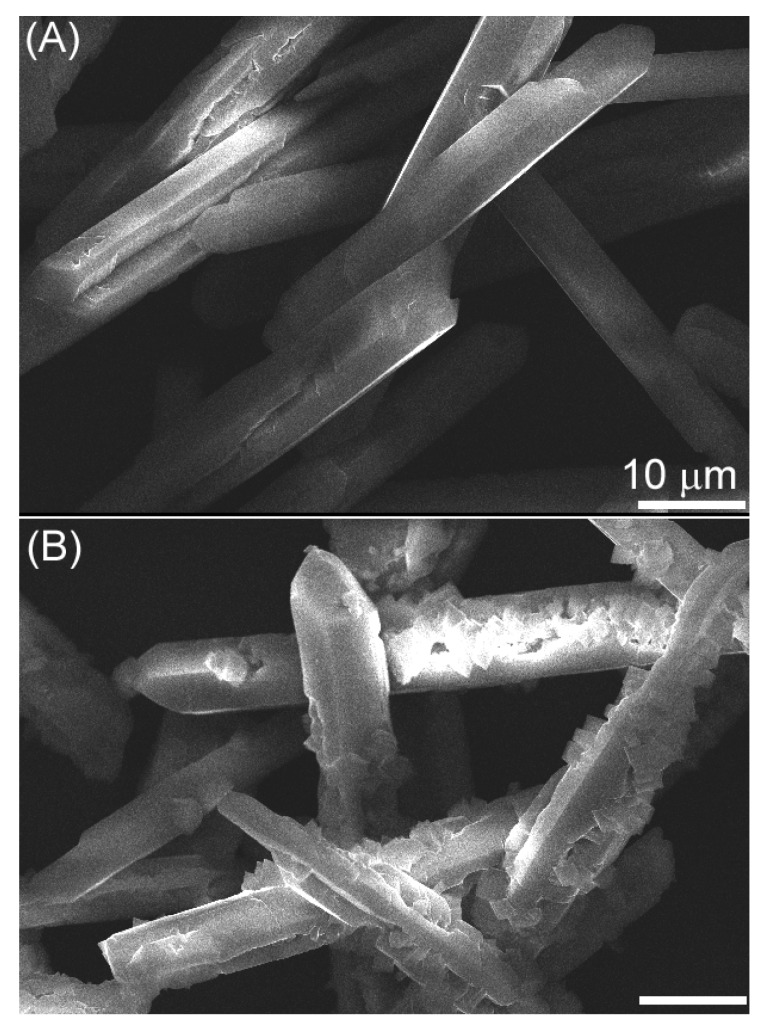
SEM images of the products obtained at different reaction times (**A**) 4 h (**B**) 10 h.

In addition, the investigation of time effect shows that eight hours is appropriate for the formation of whiskers. When the time is less than 8 h, as shown in Figure 4(A), the yield of whisker is decreased on the base of calcium source (e.g., 67.3% for 4 h), indicating that the reaction is incomplete. For longer periods of time (e.g., 10 h), no significant change occurs in the shape of products. Concave cavities (Figure 4B) appear on the surfaces of whiskers, the reason for which remains unclear.

**Figure 5:**
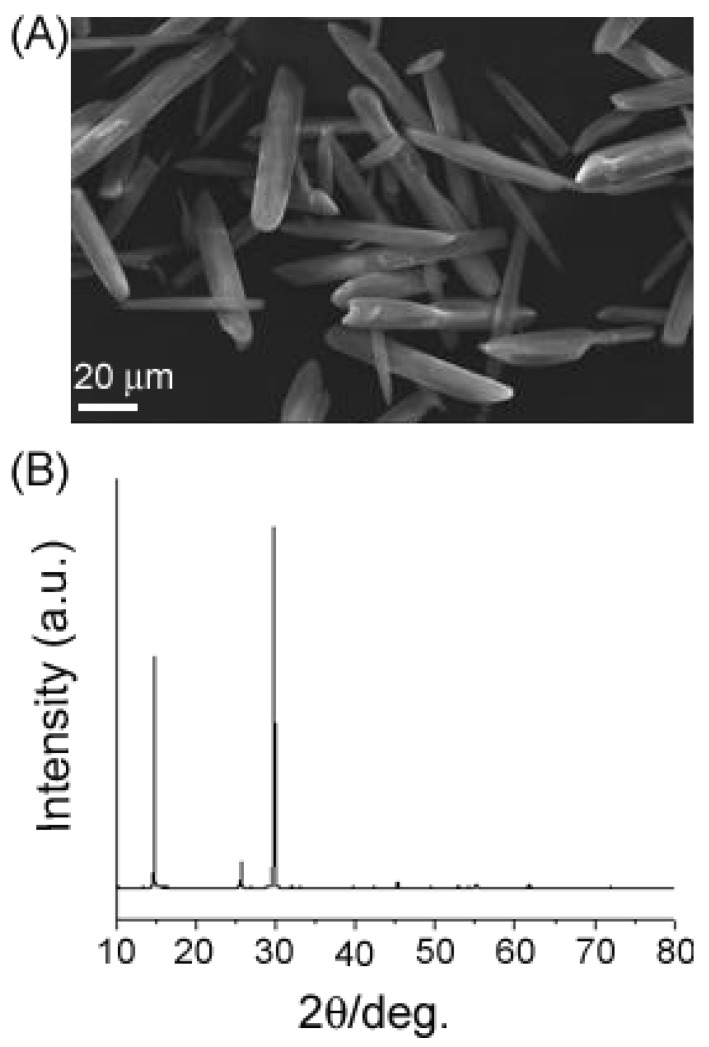
SEM image (**A**) and X-ray powder diffraction (XRD) pattern (**B**) of CaSO_4_ whiskers produced using a similar procedure.

Considering the importance of the whiskers, we try to extend the strategy to grow CaSO_4_ fibers by using CaCl_2_, (NH_4_)_2_SO_4_, and MgCl_2_ as starting materials. The SEM image and XRD pattern (Figure 5) shows that spindle-like CaSO_4_ crystalline fibers can be successfully obtained using a similar procedure. This result indicates that the present system can be generalized to synthesize other inorganic functional materials and fibers.

## 3. Experimental Section

In a standard procedure, anhydrous calcium chloride (CaCl_2_, 6.72 g), urea (NH_2_)_2_CO, 10.9 g) and magnesium chloride (MgCl_2_, 2 g) were dissolved in 42 mL of distilled water to form a homogenous solution. Then the solution was transferred to a Teflon-lined autoclave with the capacity of 50 mL and maintained at 155 °C for 6–8 h. After the reaction was complete, CaCO_3_ whiskers filled the autoclave. The whiskers were washed with distilled water several times and collected for characterization.

The X-ray powder diffraction (XRD) pattern was carried out on a Philips X’Pert PRO SUPER X-ray diffractometer equipped with graphite monochromatized Cu *Kα* radiation (λ = 0.1541874 nm). The morphology observation was performed on a polarizing microscope (POL-280-C, Beijing Maike) and Hitachi S-4800 field-emission scanning electron microscope (FE-SEM, Japan).

## 4. Conclusions

In summary, a facile hydrothermal route with the assistance of MgCl_2_ has been developed to grow well-defined tubular aragonite whiskers on a large scale. Mg^2+^ plays an important role in determining the purity and quality of the product. Meanwhile, the present route can be extended to prepare CaSO_4_ fibers, indicating that the method may be generalized to grow functional oxide materials. Of course, further studies are still necessary to provide insights into unraveling the crystal growth mechanism.
